# Dissecting Ubiquitylation and DNA Damage Response Pathways in the Yeast *Saccharomyces cerevisiae* Using a Proteome-Wide Approach

**DOI:** 10.1016/j.mcpro.2023.100695

**Published:** 2023-12-14

**Authors:** Ewa Blaszczak, Emeline Pasquier, Gaëlle Le Dez, Adrian Odrzywolski, Natalia Lazarewicz, Audrey Brossard, Emilia Fornal, Piotr Moskalek, Robert Wysocki, Gwenaël Rabut

**Affiliations:** 1 Department of Genetics and Cell Physiology, Faculty of Biological Sciences, University of Wroclaw, Wroclaw, Poland; 2 Department of Biochemistry and Molecular Biology, Faculty of Medical Sciences, Medical University of Lublin, Lublin, Poland; 3 Univ Rennes, CNRS, INSERM, Institute of Genetics and Development of Rennes (IGDR), UMR 6290, U1305, Rennes, France; 4 Department of Bioanalytics, Faculty of Biomedicine, Medical University of Lublin, Lublin, Poland

**Keywords:** DNA replication stress, Genotoxic stress, Ubiquitin proteasome system, Ubiquitination, Proteolysis, Proteostasis, Nucleus, Inner nuclear membrane, Hst3, Pol30, Erg5, Anchor-away, SILAC, NanoLuc, Yeast

## Abstract

In response to genotoxic stress, cells evolved with a complex signaling network referred to as the DNA damage response (DDR). It is now well established that the DDR depends upon various posttranslational modifications; among them, ubiquitylation plays a key regulatory role. Here, we profiled ubiquitylation in response to the DNA alkylating agent methyl methanesulfonate (MMS) in the budding yeast *Saccharomyces cerevisiae* using quantitative proteomics. To discover new proteins ubiquitylated upon DNA replication stress, we used stable isotope labeling by amino acids in cell culture, followed by an enrichment of ubiquitylated peptides and LC-MS/MS. In total, we identified 1853 ubiquitylated proteins, including 473 proteins that appeared upregulated more than 2-fold in response to MMS treatment. This enabled us to localize 519 ubiquitylation sites potentially regulated upon MMS in 435 proteins. We demonstrated that the overexpression of some of these proteins renders the cells sensitive to MMS. We also assayed the abundance change upon MMS treatment of a selection of yeast nuclear proteins. Several of them were differentially regulated upon MMS treatment. These findings corroborate the important role of ubiquitin-proteasome–mediated degradation in regulating the DDR.

## Highlights

• Proteomic discovery of DNA damage-regulated ubiquitylation sites in yeast.
• Identification of 5397 ubiquitylated peptides from 1853 proteins.
• Identification of genes whose overexpression sensitizes cells to MMS treatment.
• Identification of proteins up- and downregulated upon MMS treatment using NanoLuc.

## Graphical Abstract

**Figure:**
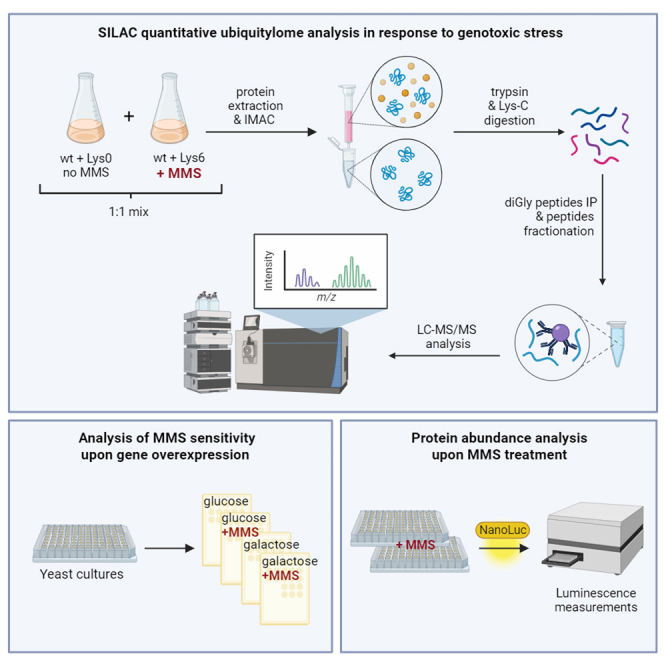

## In Brief

DNA damage response pathways (DDR) protect eukaryotic cells from the accumulation of DNA damage and genome instability, a major cause of cancer and other diseases in humans. Ubiquitylation orchestrates the regulation of DDR events. Blaszczak *et al*. use quantitative proteomics and a reporter assay based on the NanoLuc luciferase to study ubiquitylation and protein abundance changes upon genotoxic stress in yeast. The authors point out proteins that may be regulated by ubiquitylation during the DDR.

Posttranslational modification (PTM) of proteins by ubiquitin is an essential mechanism regulating a myriad of cellular processes. Ubiquitylated proteins may be directed for proteasomal degradation or can orchestrate biological processes such as cell cycle progression, transcriptional regulation, chromatin remodeling, trafficking, immune response, and the cellular response to DNA damage (1, 2, 3, 4, 5, 6). Since genomes are continuously exposed to exogenous and endogenous DNA damage, cells need to repair DNA lesions to prevent the accumulation of potentially deleterious mutations and to maintain genomic stability. Failure to repair DNA damage is the underlying cause of cancer development and premature aging in humans (7, 8). Therefore, cells evolved with a highly conserved and complex signaling network termed the DNA damage response (DDR). The understanding of the coordination of the DDR by ubiquitylation has significantly increased over the last decade (6, 6, 9, 10, 11). However, due to the complexity and versatility of both the ubiquitylation system and the DDR pathway, our comprehension of how these processes are regulated is still relatively limited.

Protein ubiquitylation involves a cascade of enzymatic reactions catalyzed by a variety of enzymes, including ubiquitin activating enzymes (E1s), ubiquitin conjugating enzymes (E2s), and ubiquitin ligases (E3s). Many of these enzymes, as well as the ubiquitin itself, are highly conserved across eukaryotes. Nuclear-localized ubiquitin ligases have been shown to regulate various DNA repair pathways both in yeast and humans (12, 13, 14). For instance, in response to replication fork stalling, proliferating cell nuclear antigen (PCNA), which serves as a replicative polymerase clamp, is monoubiquitylated at K164 by the E2 Rad6 and the E3 Rad18 to recruit the translesion synthesis polymerases that are able to replicate damaged DNA templates (15, 16, 17). Monoubiquitylated PCNA can be subsequently polyubiquitylated by the E2 Ubc13 and the E3 Rad5 (HLTF in humans) to promote the error-free DNA damage tolerance pathway that allows DNA synthesis by template switch (15, 18, 19). Another replication fork-associated E3 complex Rtt101^Mms1^ (CUL4^DDB1^ in humans) plays a role in replication through natural pause sites and progression through damaged templates (20, 21), as well as in nucleosome assembly by ubiquitylation of newly synthesized histone H3 (22, 23). CUL4^DDB1^ is also involved in PCNA monoubiquitylation at K164 (24). In budding yeast, the SCF^Cdc4^ (Skp1, Cullin/Cdc53, F-box protein Cdc4) ubiquitin ligase complex has been shown to target the sirtuin family histone deacetylase Hst3 for proteolysis (25, 26). Hst3 is responsible for the removal of K56 acetylation from newly synthesized histone H3, which has been incorporated into chromosomes during the S phase, and then Hst3 undergoes rapid degradation at the G2/M phase. In response to replication stress, Hst3 is also downregulated in a DNA damage checkpoint manner to maintain H3-K56 acetylation, which is required for proper DNA repair and cell survival (27, 28, 29, 30).

Advances in quantitative proteomics facilitated high-throughput studies of various PTMs, including phosphorylation, ubiquitylation, SUMOylation, and acetylation (31, 32, 33, 34, 35, 36, 37, 38, 39). Importantly, many of the identified PTM sites are conserved from yeast to mammals (40). The identification of ubiquitylated proteins was usually challenging due to the low abundance of ubiquitylated proteins and difficulties in the purification strategies. Progress has been made with the implementation of stable isotope labeling by amino acids in cell culture (SILAC) and ubiquitylated proteins/peptides enrichment strategies (recently reviewed by Steger and colleagues (41)). SILAC proved to be a powerful method for the relative quantification of ubiquitylated peptides and proteins (42, 43). Here, we used SILAC followed by the enrichment of ubiquitylated proteins and liquid chromatography-tandem mass spectrometry (LC-MS/MS) (44, 45) to quantitatively profile the ubiquitylome of budding yeast upon DNA replication stress induced by the DNA alkylating agent methyl methanesulfonate (MMS). We obtained a global view of protein ubiquitylation changes, providing new insight into the adaptive response to DNA damage in *Saccharomyces cerevisiae*.

## Experimental Procedures

### Yeast Strains, Plasmids, and General Growth Conditions

The *S. cerevisiae* strains and plasmids used in this study are listed in supplemental Tables S1 and S2, respectively. Yeast strains are isogenic derivatives of W303 (*MAT*a *ade2-1 can1-100 ura3-1 his3-11,15 leu2-3,112 trp1-1 RAD5*) (Rodney Rothstein) and BY4741 (*MAT*a *his3*Δ*1 leu2*Δ*0 met15*Δ*0 ura3*Δ*0*) (46). Yeast manipulations were carried out according to standard protocols (47) unless stated otherwise. Yeast cells were grown at 30 °C in standard rich medium (YPD; 1% yeast extract, 2% peptone, 2% glucose) or in synthetic complete (SC) medium (0.17% yeast nitrogen base, 0.5% ammonium sulfate, 2% glucose, supplemented with amino acids and nucleobases) (48). Overexpression plasmids were from a collection of yeast ORFs (BG1805 vector, Open Biosystems) (49). *MOT1* and *RDH54* genes were additionally cloned into the same vector. All plasmids were constructed using standard molecular biology techniques. Sequences and construction details are available upon request. Both the overexpression plasmids from the collection and newly constructed plasmids were verified by sequencing. To alleviate the toxicity that may be caused by rapamycin, anchor-away strains have the *FPR1* gene deleted and the WT *TOR1* allele replaced by the rapamycin-resistant *tor1-1* counterpart. The endogenous yeast proteins Pre8 (a subunit of the 20S catalytic core particle of the proteasome) and Sts1 (an essential protein containing a noncanonical bipartite nuclear localization signal, responsible for nuclear localization of proteasome (50)), were genetically fused with the FKBP12-rapamycin–binding (FRB) domain of human mammalian target of rapamycin (mTOR) and GFP to produce the Pre8-FRB-GFP (scGR1115) and Sts1-FRB-GFP (scGR1123) strains. *RPN7*-*tDimer2* was introduced into these strains by transformation with the linearized pRS306_*RPN7*-*tDimer2* plasmid (pGR677) bearing the *RPN7* proteasome subunit fused to the red fluorescence marker *tDimer2*. Integration of *RPN7*-*tDimer2* in the yeast genome was verified by fluorescence microscopy. The strain used for SILAC labeling (scMLB2) was derived from scGR1115 with *LYS2* deletion using standard PCR transformation and selection on α-aminoadipate plates (0.17% yeast nitrogen base, 2 g/l DL-α-aminoadipate, 30 mg/l L-lysine HCl, 2% glucose, 2% Bacto agar, supplemented with auxotrophic requirements).

### Preparation of Protein Extracts for Immunoblotting

Protein extracts were prepared as follows. The absorbance at 600 nm (A_600_) of yeast cultures (5 ml) was measured and cells were collected by centrifugation (1700*g*, 2 min). Cells were resuspended in 300 μl of 20% trichloroacetic acid (w/v) (TCA) and lysed using glass beads (Sigmund Lindner) for 2 min in a Disruptor Genie agitator (VWR). Proteins precipitated in TCA were centrifuged (21,000*g*, 5 min). TCA was carefully removed and the pellet was resuspended in a loading buffer (15% glycerol, 450 mM Tris pH 8.8, 1% SDS, 2 mM EDTA, 0.5% bromophenol blue) with the addition of 0.1 M DTT. The loading buffer volume was adjusted according to the absorbance measured initially. The samples were then denatured at 95 °C for 5 min and centrifuged (21,000*g*, 5 min). Ten microliters of each sample was added to SDS-PAGE gels.

### SDS-PAGE and Immunoblotting

Denatured protein samples and protein marker ladder (Precision Plus Protein All Blue, Bio-Rad) were run on precast 4 to 20% polyacrylamide gels (Mini-PROTEAN TGX, Bio-Rad) for 45 min at constant 150 V or 30 min at 250 V, in 25 mM Tris, 192 mM glycine, and 1% SDS running buffer. Transfer to a polyvinylidene fluoride (PVDF) membrane (Bio-Rad or Amersham) was performed using a Trans-blot Turbo transfer system (Bio-Rad) or a Power Blotter (Invitrogen) at 25 V, 1.3 A, for 7 min. Membranes were blocked in phosphate-buffered saline (PBS) containing 0.1% Tween20 (PBS-T) and 5% milk for 1 h at room temperature (RT). The membranes were then incubated overnight at 4 °C with the primary antibody in PBS-T, 5% milk. Antibodies included the following: anti-ubiquitin mouse monoclonal primary antibody (P4D1, Santa Cruz Biotechnology, at 1:1000 dilution), anti-GFP mouse primary antibody (clones 7.1 and 13.1, Roche, at 1:2000 dilution), anti-ß-actin mouse monoclonal primary antibody (Invitrogen, at 1:2000 dilution), and anti-K48-linked ubiquitin primary antibody (clone Apu2, Merck Millipore, at 1:1000 dilution). Next, membranes were washed three times, incubated for 45 min with appropriate secondary antibody coupled to horseradish peroxidase diluted in PBS-T, 5% milk and rewashed again three times prior to development. The membranes were then incubated with enhanced chemiluminescence substrates (either SuperSignal West Dura Extended Duration Substrate or 100 mM Tris pH 8.5, 1.25 mM luminol, 0.2 mM coumaric acid, 0.01% hydrogen peroxide) and revealed on photographic films (Midsci) with a Agfa Curix 60 film processor or using a digital imager (Amersham Imager 680).

### Microscopy of Living Yeast Cells

Analysis of cellular localization of fluorescent proteins (GFP and red fluorescent protein, tDimer2) was performed in living cells. Yeast cells were allowed to sediment in Lab-Tek chambers (Nunc) and maintained at 30 °C during the entire image acquisition process. At point t_0_, rapamycin (LC Laboratories) was added into the wells at 1 μg/ml final concentration. Images were acquired using a NIKON Eclipse Ti-E inverted microscope (Scientifica) equipped with a 100× oil immersion objective (NA 1.5), a green and red filter set and DIC. Images were processed using ImageJ software (https://imagej.net/ij/).

### Yeast Immunofluorescence

Yeast cultures were fixed with 4% formaldehyde for 1 h at 30 °C. The cells, recovered by centrifugation, were washed in an isotonic buffer (0.1 M phosphate pH 6.6, 1 M sorbitol). After two washes, the yeast cell wall was digested for 1 h by adding zymolyase 20T (AMSBIO) at 150 μg/ml. The cells were recovered by centrifugation, resuspended in the isotonic buffer, and sedimented for 30 min on a coverslip pre-incubated with 0.1% polylysine. Adherent cells were permeabilized (10 min in PBS containing 1% Triton X-100) and blocked (10 min in PBS containing 5 mg/ml bovine serum albumin (PBS-BSA)). The coverslips were then sequentially incubated for 1 h with the primary antibody (clone Apu2, Merck Millipore, 1:500 dilution in PBS-BSA), washed four times with PBS-BSA, incubated for 45 min in the dark with the secondary antibody coupled to Alexa Fluor 546 and 4′,6-diamidino-2-phenylindole at 1 μg/ml, washed four times with in PBS-BSA, washed once with water, and mounted in Mowiol 4-88 (Sigma-Aldrich). Image acquisition was performed with a NIKON Ti-E microscope (Scientifica) equipped with a 100× oil immersion objective (NA 1.5).

### SILAC and MMS Treatment

The scMLB2 strain (auxotroph for lysine) was transformed with a plasmid carrying N terminally His-tagged ubiquitin (pGR295) (51). Yeast cells were grown in a SC medium (without lysine and leucine) supplemented with either “light” lysine (l-lysine ^12^C_6_^14^N_2_, Sigma-Aldrich, L-1262) or “heavy” lysine (l-lysine ^13^C_6_^14^N_2_, Sigma-Aldrich, 643459) at 10 mg/l. Cultures were grown for 3 days at 30 °C with shaking at ≥200 rpm. Each day, cultures were diluted and supplemented with fresh lysine for continuous incorporation. During the third day, cells were diluted and treated with rapamycin at 1 μg/ml final concentration for 5 h (cells maintained in log-phase at the end of the treatment). In the last hour of incubation with rapamycin, MMS (Sigma-Aldrich) was added to cells cultured with “heavy” lysine at a final concentration of 0.02% and the cells were cultivated for an additional hour at 30 °C with agitation.

### Preparation of Protein Extracts for Mass Spectrometry

Cells cultured with and without MMS were mixed in 1:1 ratio and harvested by centrifugation using a JLA9.1000 rotor (Beckman Coulter) for 3 min at 12,000*g*. After supernatant removal, the cell pellet was transferred into a 50 ml syringe, and cells were slowly added dropwise to the liquid nitrogen in a glass beaker. Frozen cell droplets were weighted and 5 g were ground under cryogenic conditions using a CryoMill homogenization device (Retsch). Ground cells were resuspended in 20 ml of 20% TCA (4 °C), split into 5 ml tubes and centrifuged at 21,000*g* for 5 min at 4 °C. Next, 4 ml of 20% TCA was added to each tube, and pellets were resuspended and centrifuged as above. TCA was carefully removed. The pellets were resuspended in 20 ml of purification buffer (6 M guanidine-HCl, 100 mM Tris pH 9, 300 mM NaCl, 0.2% Triton X-100, 5 mM chloroacetamide, 10 mM imidazole; pH 8.5) to a 50 ml falcon tube and solubilized on a rotating wheel for 10 min at RT. The suspension was centrifuged at 21,000*g* for 30 min at 4 °C to remove the cellular debris. Protein concentration in the total extract was measured using a bicinchoninic acid protein assay (Pierce BCA Protein Assay Kit, Thermo Fisher Scientific) at 562 nm according to the manufacturer’s protocol.

### Enrichment of Ubiquitylated Proteins from the Lysate by Immobilized Metal Affinity Chromatography

Four milligrams of total proteins were purified using TALON Metal Affinity Resin (Clontech), which was equilibrated with the purification buffer. Ubiquitylated proteins were allowed to bind for 1 h 30 min at RT on the rotating wheel. Then, nonbound proteins were removed by centrifugation at 1700*g* for 5 min, the resins were washed twice with wash buffer I (8 M urea, 100 mM Na-phosphate buffer pH 7, 300 mM NaCl, 0.2% Triton X-100, 5 mM chloroacetamide, 5 mM imidazole; pH 7) for 10 min and twice with wash buffer II (8 M urea, 100 mM Na-phosphate buffer pH 7, 300 mM NaCl, 0.2% Triton X-100, 5 mM chloroacetamide, 0.2% SDS, 5 mM imidazole; pH 7) for 10 min. The beads were transferred to a Costar (Thermo Fisher Scientific) chromatography column and the bound proteins were eluted in 300 μl of elution buffer (6 M urea, 50 mM Tris–HCl pH 6.8, 300 mM NaCl, 0.2% Triton X-100, 5 mM chloroacetamide, 0.2% SDS, 250 mM imidazole; pH 8). Ten microliters of the sample was run on polyacrylamide 4 to 20% SDS-PAGE gel (Mini-PROTEAN TGX, Bio-Rad) and affinity-purified proteins were verified by silver staining. Briefly, the gel was fixed in 10% acetic acid and 40% ethanol and subsequently incubated with 50% and 30% ethanol for 20 min with shaking. Then, it was incubated in 0.8 mM Na_2_S_2_O_3_ for 60 s and washed three times with water. The gel was impregnated with AgNO_3_ (2 g/l) with an addition of 0.026% formaldehyde. After washing three times with water, the proteins in the gel were visualised in 3% Na_2_CO_3_, 0.0185% formaldehyde, and 16 μM Na_2_S_2_O_3_. The reaction was stopped with 10% acetic acid.

### Lysyl Endopeptidase and Trypsin Digestion

Purified proteins were digested, first with 20 μg lysyl endopeptidase (Lys-C) (Wako) for 3 h at 20 °C, diluted in 50 mM Tris pH 8, and then with 20 μg of trypsin (Pierce trypsin protease, mass spectrometry (MS) grade, Thermo Fisher Scientific) overnight at 20 °C. A mixture of peptides was then acidified with 0.5% (final concentration) of formic acid (FA) and subsequently desalted using a SEP-PakVac tC_18_ cartridge column (Waters). C_18_ cartridges were conditioned with 0.5 ml of 100% acetonitrile (ACN), followed by 0.5 ml of 50% ACN and 0.1% FA, and finally ∼1.2 ml of 0.1% trifluoroacetic acid (TFA). Digested samples were loaded onto the conditioned C_18_ cartridge column, washed twice with 1 ml of 0.1% TFA, and eluted with 600 μl of 50% ACN and 0.1% FA. Eluates were lyophilized at −80 °C using Heto Drywinner (Thermo Fisher Scientific).

### K-ε-GG Peptide Enrichment: Antibody Crosslinking and Immunoaffinity Purification

Anti-K-ε-GG antibody was obtained as part of the PTMScan ubiquitin remnant motif (K-ε-GG) kit (Cell Signaling Technology). Immunoaffinity purification of ubiquitylated peptides was carried out according to the manufacturer’s instructions with some modifications. In brief, ∼250 μg of antibody-coated beads were resuspended in 0.5 ml of sodium borate (100 mM, pH 9), transferred to a Costar chromatography column (Thermo Fisher Scientific) and rotated 5 min on a wheel at RT. Next, the beads were centrifuged 2 min at 2000*g* at 4 °C, washed twice with sodium borate and centrifuged as above. For antibody crosslinking, 0.5 ml of a dimethyl pimelimidate containing solution (20 mM dimethyl pimelimidate dissolved in 100 mM sodium borate, pH 9) was added to the column and the beads were rotated on the wheel 30 min at RT. The crosslinking reaction was stopped and the unreacted sites were blocked by washing the beads twice with 0.5 ml ethanolamine (200 mM, pH 8) and overnight incubation at 4 °C on a rotating wheel. The crosslinked beads were then washed three times 5 min with 0.5 ml of ice-cold immunoaffinity purification buffer (50 mM 3-(N-morpholino)propanesulfonic acid pH 7.2, 10 mM sodium phosphate, 50 mM NaCl). Lyophilized peptides were resuspended in 0.5 ml of 1x ice-cold immunoaffinity purification buffer and incubated overnight with the crosslinked anti-K-ε-GG beads on a rotating wheel at 4 °C. Beads were finally washed three times with 0.5 ml of ice-cold PBS and three times with 0.5 ml of ice-cold water prior to elution of enriched ubiquitylated peptides with 100 μl of 0.15% TFA solution.

### LC-MS/MS Analysis

To increase the number of peptides identified during LC-MS/MS analysis, peptide fractionation was performed by reversed-phase chromatography (Pierce High pH Reversed-Phase Peptide Fractionation Kit; Thermo Fisher Scientific). Eight fractions were collected according to the manufacturer’s instructions. One microgram of peptides from each fraction was subjected to LC-MS/MS analysis. Analyses were performed on an Orbitrap Fusion mass spectrometer equipped with an easy spray ion source and coupled to an EASY nano-LC 1200 instrument (Proxeon, Thermo Fisher Scientific) at the Proteomic Platform of the Institute Jacques Monod. The peptides were loaded using an on-line sample preconcentration method and separated using a mobile phase consisting of 0.1% FA in water (solvent A) and 0.1% FA in 80% v/v ACN-water (solvent B) on an Acclaim Pepmap RSLC C18 column (0.75 mm × 750 mm, 2 μm, 100 Å; Thermo Fisher Scientific). Beforehand, the column was equilibrated with 95% solvent A for 5 min. Then, the solvent B was increased to 28% in 105 min, and to 40% in 15 min. The gradient was followed by column washing with 95% of B for 20 min, and column re-equilibrating at 95% of A for 10 min. The system operated at a nano-flow rate of 300 nl/min and the column temperature was set at 50 °C. The advanced peak determination algorithm was used during the acquisition to increase the number of precursors subjected to a collision cell for the fragmentation. The Orbitrap Fusion mass spectrometer operated in full ion scan mode, at a resolution of 120,000 and a capillary voltage of 2.1 kV. Data were acquired from 350 to 1550 *m/z*. The automatic gain control target was set at 4 × 10^5^ and 1 × 10^4^ for MS and tandem mass spectrometry (MS/MS), respectively. For MS/MS experiments the 3 s mode was employed. Peptide fragments were generated *via* higher-energy C-trap dissociation with the normalized collision energy value of 27% and the dynamic exclusion of 60 s. Monocharged peptides and those of unassigned charge states were excluded from the acquisition. One hundred milliseconds for MS and 35 ms for MS/MS, respectively, were set as the maximum accumulation times. The raw mass spectrometric data (LC-MS/MS–based quantitation) were processed using Mascot peptide search engine (v 2.5.1) and Proteome Discoverer (v 2.5.0.400) software (Thermo Fisher Scientific).

### Protein and Peptides Identification

MS/MS spectra were extracted and searched using the Mascot peptide search engine (v 2.5.1). Search parameters were set as 10 ppm mass tolerance on the precursor ion and 0.8 Da tolerance on fragment ions. The minimum precursor mass was set to 350 Da and the maximum precursor mass was set to 5000 Da. Tandem mass spectra were matched with possible peptide sequences. The minimum peptide length was set to 6. The UniProtKB/Swiss-Prot database was used to retrieve yeast protein sequences (*S. cerevisiae*, strain ATCC 204508/S288c, accessed: October 2017, 7909 entries), including decoy-reversed sequences and the common contaminants. The false discovery rate (FDR) for both peptide and protein identification was 1%. Methionine oxidation (a monoisotopic mass of 15.99 Da), ubiquitin diglycine remnant (−GG signature; a monoisotopic mass of 114.04 Da), and Leu-Arg-Gly-Gly remnant (−LRGG signature generated by one missed trypsin cleavage; a monoisotopic mass of 383.23 Da) were set as variable PTMs. Fixed PTM included carbamidomethylation on cysteine residues (a monoisotopic mass of 57.02 Da). The maximum of PTMs per peptide was set to 10. Thermo Proteome Discoverer (v 2.5.0.400) was used for further peptide, protein and modification analysis, and additional R-scripts (v. 4.1.2) were used for combining and plotting the data.

### Gene Ontology–Based Functional Analysis

R (v. 4.1.2) was used to perform gene ontology (GO)-based functional analysis. The intersection between upregulated genes and selected ontologies (supplemental Table S5) was done based on org.Sc.sgd.db (v. 3.14.0) database using tidyverse (v. 1.3.2) and AnnotationDbi (v. 1.56.2). Results were presented using the ggplot2 (v. 3.4.0) and ggrepel (v. 0.9.3) packages.

### Spot Assays

To assay the MMS sensitivity of yeast cells transformed with plasmid-bearing genes of interest under the control of *GAL1* promoter, yeast cells were grown overnight at 30 °C in liquid minimal medium (0.17% yeast nitrogen base, 0.5% ammonium sulfate, 2% raffinose as a carbon source) supplemented with the only necessary amino acids and nucleobases and without uracil. The *A*_600_ of the cultures was measured and adjusted to 0.3. Then, 10-fold serial dilutions of cell suspensions were prepared in water. Two microliters of each dilution was spotted using a multipin apparatus (for square plates) or using a pipet (for round plates) on solid minimal media (without uracil) containing glucose or galactose as a carbon source with or without 0.012% MMS. Growth was monitored after 3 to 4 days at 30 °C. Plates were imaged using ChemiDoc Imaging System (Bio-Rad).

### NanoLuc Reporter Assay

We created a library of yeast strains expressing proteins endogenously fused to the NanoLuc luciferase (unpublished). We have selected yeast strains from this library to analyze the abundance of the corresponding NanoLuc-tagged proteins upon MMS treatment. The selected strains were then handled using a microbial pinning robot (ROTOR HDA, Singer). Cells were grown in a 96-well plate overnight in SC medium (without leucine) at 30 °C. Overnight cultures were diluted to an *A*_600_ of 0.2 and grown for another 5 h in two 96-well plates so that the measurements could be performed in duplicate for each protein. NanoLuc substrate furimazine was diluted 1/1000 (5 μM final concentration) in the culture medium. Twenty microliters of each culture was added onto white 96-well half-area microplates (Greiner), followed by 20 μl of the diluted furimazine. MMS was then added into each well at a final concentration of 0.03% and the plates were mixed thoroughly. Luminescence measurements were performed at 10 min and 2 h after MMS addition using an EnSight multimode plate reader (PerkinElmer).

### Experimental Design and Statistical Rationale

The SILAC experiment was performed as a single replicate with two independent cultures (untreated and MMS-treated, labeled with “light” or “heavy” lysine, respectively) mixed in a 1:1 ratio and treated as described in the method section to produce eight peptide fractions, which were then analyzed by the LC-MS/MS. A limitation of this experimental design is that it does not permit evaluating the experimental variability of the peptide heavy-to-light (H/L) abundance ratios. Yet, we evaluated the significance of these ratios by two methods. First, we used the Proteome Discoverer software to determine whether the H/L ratio of any given peptide is significantly different from the H/L ratio of the other quantified peptides (background-based ANOVA with Benjamini–Hochberg correction for FDR). This method does not require experimental replicates, but it does require that most of the peptide abundances are unchanged between both conditions (MMS-treated and MMS-untreated). In addition, we used the significance B module of the Perseus software (v2.0.11) (https://maxquant.net/perseus/) (52), which calculates *p*-values for the detection of significant H/L ratios using subsets of peptides grouped by intensity binning (53) (the FDR for Benjamini–Hochberg correction was set to 0.05). The *p*-values computed with both methods for all K-ε-GG peptides are provided together with H/L ratios and further peptide information in supplemental Table S3). Spot assays were performed with three biological replicates and a representative image is shown. The experiments with NanoLuc were performed in at least three biological replicates (independent experiments carried out on different days), with measurements of each investigated strain in duplicates. The results of each experiment were normalized for the number of cells (*A*_600_) and log_2_ transformed. The obtained data are presented as the mean ± SD. Statistical analysis was carried out using GraphPad Prism 8.0 software (https://www.graphpad.com/) (ordinary one-way ANOVA; statistical significance: ∗∗∗∗ *p* < 0.0001, ∗∗∗ *p* < 0.0002, ∗∗ *p* < 0.0021, ∗ *p* < 0.00332).

## Results

### Specific Inactivation of Nuclear Proteasomes Using the Anchor-Away Technique

**Fig. 1:**
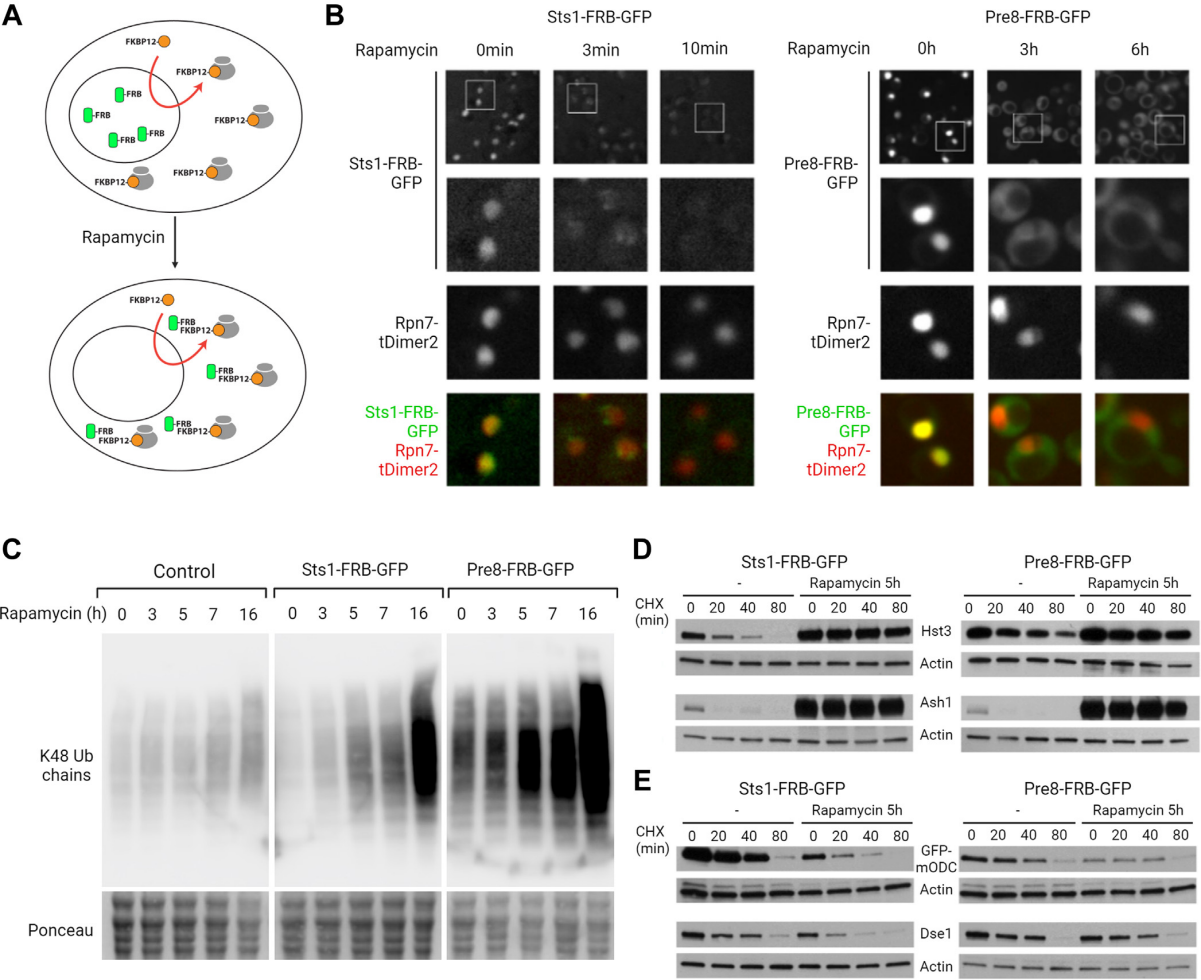
**The anchor-away strategysuppresses nuclear proteasome activity.***A*, the anchor-away strategy relies on the conditional interaction between FKBP12 and FRB (FKBP12-rapamycin-binding domain of human mTOR), which is induced by rapamycin. FKBP12 is fused to an abundant anchor protein (here, the ribosomal RPL13A), while FRB is fused to a target protein of interest. In the presence of rapamycin, the target protein is sequestered by the anchor protein. *B*, the anchor-away strategy was used to deplete nuclear proteasomes. The proteins Pre8 (a subunit of the 20S proteasome) and Sts1 (a protein required for localizing proteasomes to the nucleus) were chromosomally tagged with FRB and GFP. Additionally, the Rpn7 proteasome subunit was fused with the red fluorescent protein tDimer2. The localization of Pre8-FRB-GFP and Sts1-FRB-GFP was observed at different time points after the addition of rapamycin. *C*, K48-linked ubiquitin chains accumulate after rapamycin treatment in the Pre8-FRB-GFP and Sts1-FRB-GFP strains. The parental anchor away strain (HHY168) was used as a control. Ponceau staining was used to control loading. *D*, nuclear proteasome activity is reduced in rapamycin-treated Pre8-FRB-GFP and Sts1-FRB-GFP cells. Pre8-FRB-GFP and Sts1-FRB-GFP strains transformed with plasmids expressing the tagged nuclear proteins Hst3 and Ash1 were treated with cyclohexamide (CHX, 100 μg/ml) for the indicated times. After CHX addition, cells were lysed and analyzed by SDS-PAGE and immunoblotting. In rapamycin-treated cells, Hst3 and Ash1 proteins accumulate and are not degraded. Actin was used as a loading control. *E*, cytoplasmic proteasome activity is maintained in rapamycin-treated Pre8-FRB-GFP and Sts1-FRB-GFP cells. Pre8-FRB-GFP and Sts1-FRB-GFP strains transformed with plasmids expressing the tagged cytoplasmic proteins GFP-mODC and Dse1 were treated with CHX (100 μg/ml) for the indicated times. After CHX addition, cells were lysed and analyzed by SDS-PAGE and immunoblotting. GFP-mODC and Ash1 are degraded with and without the addition of rapamycin. Actin was used as a loading control.

To identify nuclear proteins that are ubiquitylated and degraded after DNA damage, we devised a strategy to specifically deplete nuclear proteasomes using the anchor-away technique (54). The principle of this strategy is presented in Figure 1*A*. Briefly, we tagged the ribosomal protein RPL13A with FKBP12 (the human cytoplasmic FK506-binding protein) and the proteasome subunit Pre8 or the proteasome nuclear import adapter Sts1 with the FKBP12-rapamycin–binding (FRB) domain of human mTOR. Rapamycin binds to FKBP12, which creates a surface for the interaction with FRB. Hence, FKBP12- and FRB-tagged proteins bind strongly to each other upon rapamycin treatment, forming a tight ternary complex. As a consequence, ribosome subunits synthesis and nuclear export drive Pre8 or Sts1 out of the nucleus, which—we expect—should lead to a depletion of nuclear proteasomes.

Here, we show that the anchor-away strategy specifically suppresses nuclear proteasome activity (Fig. 1, *B*–*D* and supplemental Fig. S1). The Pre8-FRB-GFP and Sts1-FRB-GFP are both depleted from the nucleus upon rapamycin treatment (Fig. 1*B*). Nuclear depletion of the Pre8-FRB-GFP is slow. It is observable after 1 h 30 min of rapamycin treatment (not shown) and requires 6 h to reach its maximum. In contrast, the Sts1-FRB-GFP is quickly depleted from the nucleus upon rapamycin treatment. Its relocation to the cytoplasm occurs within 5 min of treatment and is completed in less than 10 min. Moreover, we observed an accumulation of the K48-linked ubiquitin chains upon rapamycin treatment in both Pre8-FRB-GFP and Sts1-FRB-GFP strains (Fig. 1*C*). This accumulation occurred at a similar rate in both strains. It was clearly visible 5 h after rapamycin addition and reached its maximum at 16 h. Furthermore, immunofluorescence staining showed that K48-linked ubiquitin accumulation occurred in the nucleus (supplemental Fig. S1). Importantly, only marginal accumulation could be detected in the parental anchor-away strain that does not express FRB-tagged proteins (Fig. 1*C* and supplemental Fig. S1), indicating that the accumulation of K48-linked ubiquitin chains is primarily caused by the depletion of nuclear proteasomes. We then analyzed the turnover of nuclear and cytoplasmic proteins 5 h after rapamycin treatment using a cycloheximide chase assay (Fig. 1, *D* and *E*). To this end, we selected two nuclear proteins, Hst3 and Ash1, and two cytoplasmic protein, Dse1 and the synthetic protein GFP-mODC, all of which are rapidly degraded. The half-life of these proteins upon addition of cycloheximide (a ribosomal inhibitor, which therefore blocks protein synthesis) was determined in the Pre8-FRB-GFP and Sts1-FRB-GFP strains after 5 h of culture with or without rapamycin. In both strains, the nuclear proteins Hst3 and Ash1 are efficiently degraded in the absence of rapamycin, whereas they are no longer degraded and accumulate in the presence of rapamycin (Fig. 1*D*). The cytoplasmic proteins, Dse1 and GFP-mODC, are degraded in the absence and presence of rapamycin in both strains (Fig. 1*E*). These results indicate that after 5 h of treatment with rapamycin, the proteasome is strongly inactivated in the nucleus, while remaining active in the cytoplasm. Altogether, these results demonstrate that anchor-away–based depletion of nuclear proteasomes is efficient.

### Global Profiling of Ubiquitylation in Response to MMS

**Fig. 2:**
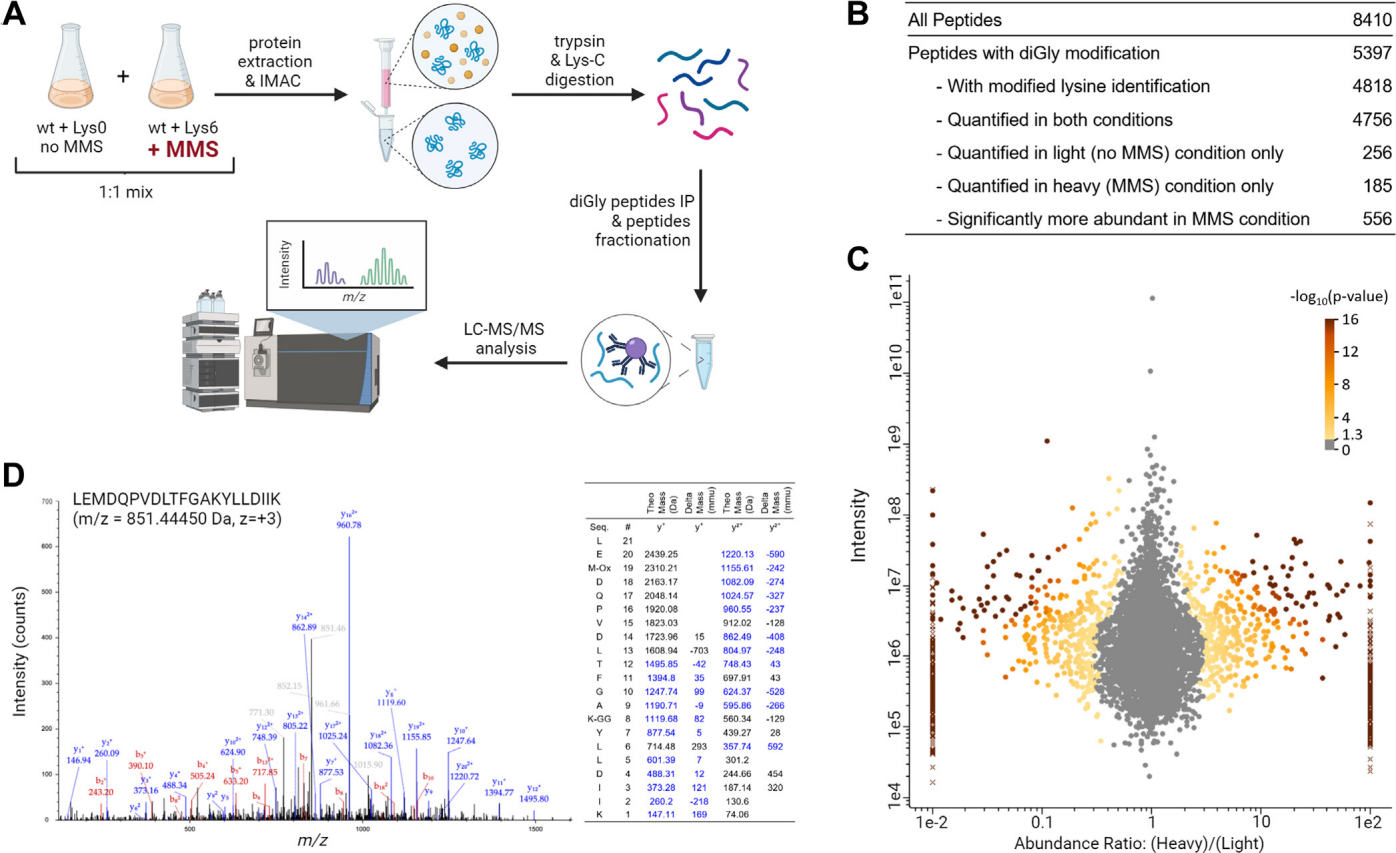
**Global profiling of ubiquitylation in response to MMS in yeast.***A*, workflow of the SILAC-based quantitative proteomic approach. Pre8-FRB-GFP anchor-away cells expressing His-tagged ubiquitin were cultured in “light” (labeled with Lys0) and “heavy” (labeled with Lys6) SILAC medium. Both cultures were subjected to rapamycin treatment for 5 h. In the last hour of the rapamycin treatment, MMS was added to the “heavy” culture at a final concentration of 0.02% (+ MMS). Subsequently, both cultures were mixed (in a 1:1 ratio), proteins were extracted and ubiquitylated proteins were purified using immobilized metal affinity chromatography (IMAC). Proteins were then digested with Lys-C endopeptidase and trypsin. The resulting peptides were immunoprecipitated with anti-ubiquitin remnant motif (K-ε-GG) antibody and fractionated. The peptides were analyzed with LC-MS/MS and heavy-to-light ratios were quantified. *B*, overview of the identified peptides. *C*, scatter plot representing the heavy-to-light ratios (*x-*axis, log_10_ scale) and intensities (*y-*axis, log_10_ scale) of all identified K-ε-GG peptides. Data from peptides quantified in both conditions are indicated with filled *circles.* Data from peptides quantified in one condition only are indicated with diagonal *crosses*. All data points are colored based on the significance of the heavy-to-light ratio (-log_10_(*p*-value)). *D*, MS/MS spectrum for a peptide corresponding to Pol30 with ubiquitin remnant motif at K210. A table with the theoretical mass and the delta mass of *y*^*+*^ and *y*^*++*^ ions is provided next to the spectrum. SILAC, stable isotope labeling with amino acids in cell culture.

**Fig. 3:**
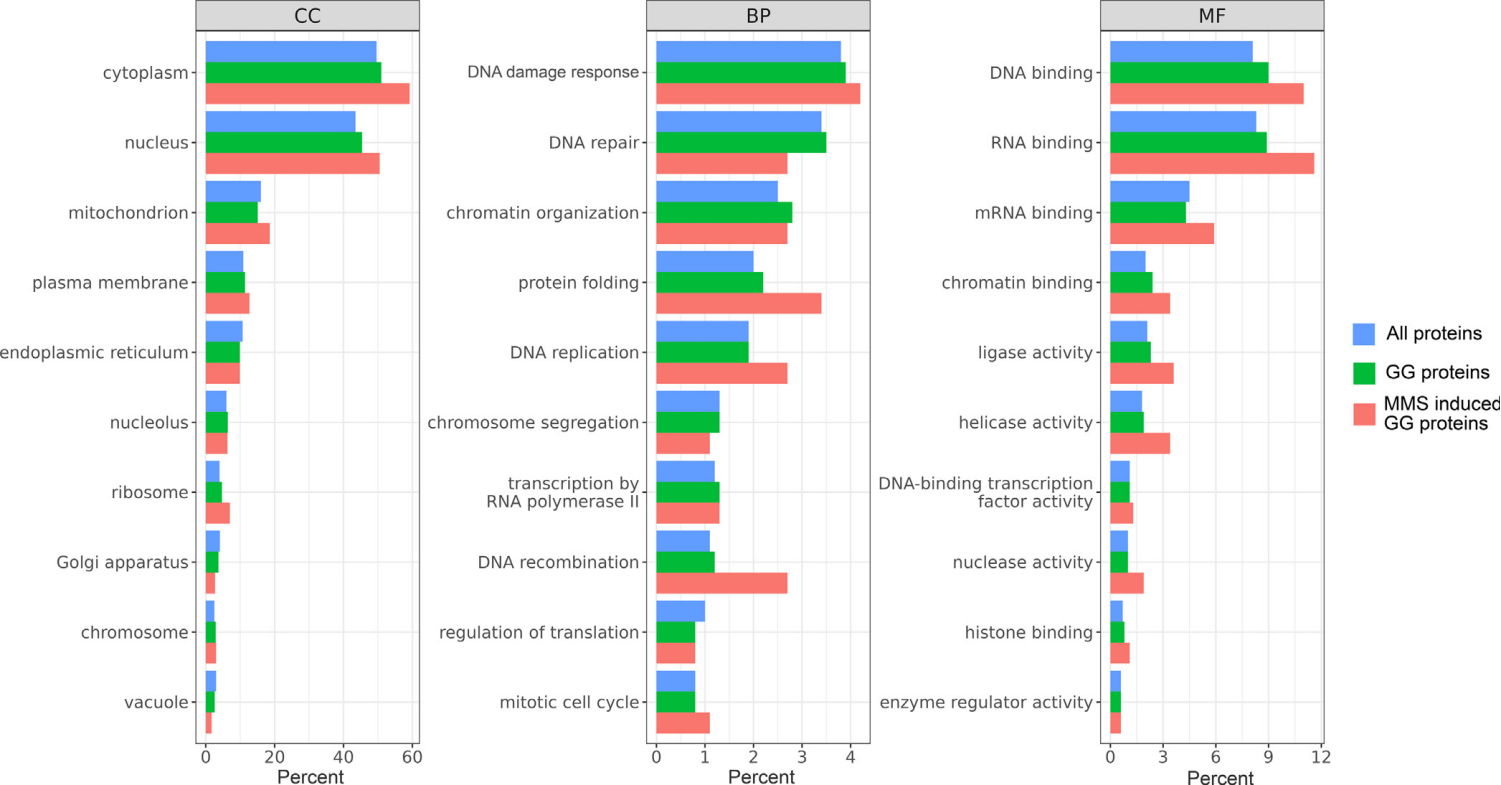
**Gene ontology analysis of proteins identified by LC-MS/MS.** Frequency of proteins annotated with selected GO slim terms from the categories: “cellular component” (CC), “biological process” (BP), and “molecular function” (MF). The analysis was performed for all proteins identified by LC-MS/MS (All proteins, *blue*), proteins with at least one K-ε-GG peptide (GG proteins, *green*), and proteins with at least one K-ε-GG peptide significantly enriched in MMS treated cells (MMS-induced GG proteins, *red*). The *top* ten most abundant GO terms from each category are presented. The selection of GO terms used for this analysis is available in supplemental Table S5, and the percentage of annotated proteins with the exact numbers for each category is available in supplemental Table S6. GO, gene ontology; MMS, methyl methanesulfonate.

We then aimed to profile ubiquitylation in response to genotoxic stress induced by MMS. The proteomic strategy we used combined metabolic labeling and immunoaffinity purification of peptides modified by a diGlycine remnant motif (K-ε-GG peptides), which are generated by Lys-C and trypsin digestion of ubiquitylated proteins (31, 55, 56, 57, 58, 59, 60) (Fig. 2*A*). We modified the previously described Pre8-FRB-GFP anchor-away strain to allow metabolic labeling of lysine residues. Further, in order to maximize the amount of K-ε-GG peptides that could be loaded and recovered during the affinity purification step, we expressed His-ubiquitin in this strain, allowing the purification of ubiquitylated proteins under denaturing conditions prior protein digestion. Expression of this exogenous form of ubiquitin had little or no effect on the ubiquitin profile (as judged by immunoblotting of total and K48-linked ubiquitin) and the MMS sensitivity of this strain (supplemental Fig. S2). Two cultures were grown with “light” and “heavy” lysine–containing medium, and nuclear proteasomes were depleted by rapamycin treatment for 5 h. Cells cultured with “light” lysine were left untreated, while cells cultured with “heavy” lysine were further treated by MMS (0.02%) to trigger the DDR. Using LC-MS/MS, we quantified 5397 K-ε-GG peptides corresponding to 1853 proteins (Fig. 2, *B* and *C* and supplemental Table S3, data available *via* ProteomeXchange with identifier PXD043291). Among these, 556 peptides from 473 proteins were upregulated more than 2-fold in response to MMS treatment (log_2_ (H/L) peptide ratio >1; *p*-value <0.05). Conversely, 644 peptides from 513 proteins were downregulated (log_2_ (H/L) peptide ratio <−1; *p*-value <0.05). The peptides significantly upregulated in response to MMS enabled us to localize 519 ubiquitylated residues in 435 proteins, among which 260 (50%) are not referenced in the BioGRID database (supplemental Table S4). Based on these data, we identified several ubiquitylation sites from proteins already known to be ubiquitylated upon the DDR. For instance, we identified two peptides corresponding to the well-known and conserved K164 and K168 ubiquitylation sites of Pol30 (the yeast PCNA protein) (15, 16, 34, 61, 62, 63) ([H/L] ratios of 8.664 and 3.316, *p*-value <0.05). Interestingly, we also detected K210 as a new ubiquitylation site of Pol30 upon MMS treatment (H/L ratio of 4.077, *p*-value <0.05) (Fig. 2*D*). However, we did not capture ubiquitylation sites for Cdc25, whose human homologs (CDC25A, CDC25B) are also known to be ubiquitylated and degraded in response to DNA damage (64, 65). The histone deacetylase Hst3, which is well known to be rapidly degraded upon MMS (26), was also not captured using this approach. Interestingly, we found a more than 50-fold increase in response to MMS for a peptide with a novel ubiquitylation site corresponding to Hst4, a paralog of Hst3. We also identified novel sites on peptides corresponding to proteins potentially ubiquitylated upon MMS, including DNA polymerase components (*e.g.*, Pol1, Pol12, Pol32), proteins involved in DNA replication (*e.g.*, Mcm3, Mcm7, Orc3), DNA repair (*e.g.*, Rad51, Rfa2, Rdh54), and transcription (*e.g.*, Ssl1, Rpa135, Cdc73, Mot1, Dal80). To determine whether proteins involved in certain biological activities were more prone to be ubiquitylated upon MMS treatment, we then performed functional analysis of MMS-regulated ubiquitylated proteins (log_2_ (H/L) ratio >1, *p*-value <0.05). We selected GO terms for subcellular localization, biological processes and molecular functions (supplemental Table S5) and compared the frequency of proteins annotated with these terms between three populations of proteins: all proteins identified in our LC-MS/MS analysis; proteins matched with at least one K-ε-GG peptide (GG proteins); and proteins matched with at least one K-ε-GG peptide upregulated upon MMS (MMS-induced GG proteins) (Fig. 3). For most GO terms, the percentage of annotated proteins (supplemental Table S6) was very similar among these three populations. Although proteins ubiquitylated upon MMS had a tendency to be more frequently annotated with GO terms related to DNA transactions (*e.g.,* DNA recombination, DNA replication, DNA binding), the enrichments were not statistically significant. There was also no enrichment for the GO term “nucleus” despite the inactivation of nuclear proteasomes with the anchor-away strategy. This result suggests that MMS-regulated ubiquitylation events are involved in diverse biological activities that may or may not be directly related to the DDR.

### Analysis of MMS Sensitivity Upon Gene Overexpression

**Fig. 4:**
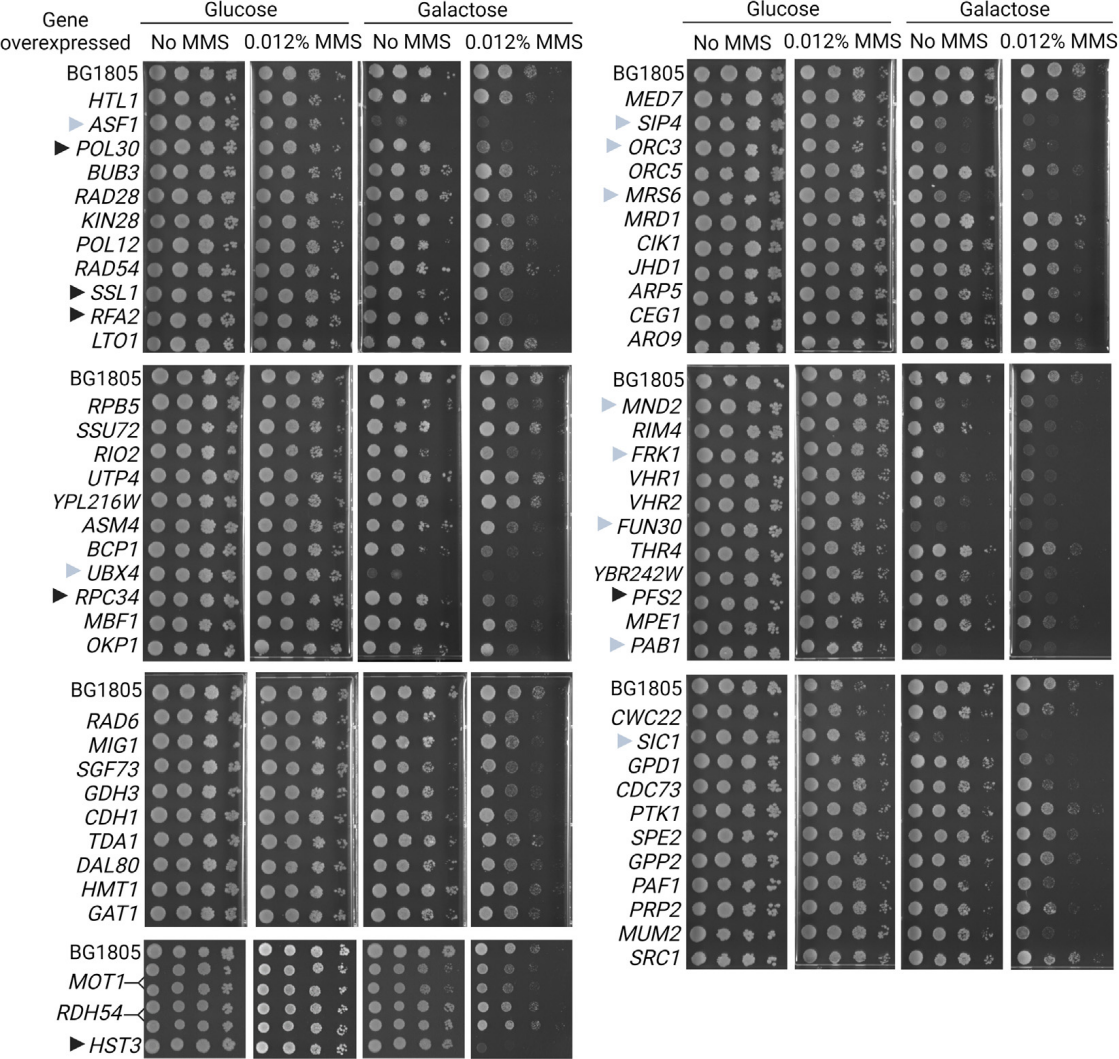
**Effect oftheoverexpression of selected genes on MMS tolerance**. Yeast cells were transformed with plasmids bearing the indicated genes expressed under the control of the *GAL1* promoter. Cultures of the transformants were then serially diluted and spotted on minimal media containing either glucose or galactose as a carbon source and supplemented with or without MMS (0.012%). Strains bearing *MOT1* and *RDH54* overexpression plasmids were spotted in duplicates. Strain bearing an *HST3* overexpression plasmid and an empty plasmid (BG1805) were used as controls. *Black triangles* indicate some genes whose overexpression induces a visible MMS-dependent growth inhibition effect; *gray triangles* indicate genes whose overexpression induces a prominent growth inhibition effect in the absence of MMS.

Since we were particularly interested in nuclear ubiquitylation, we chose a pool of 66 LC-MS/MS–detected proteins, residing in the nucleus or the nucleus and the cytoplasm, to further analyze the role of their ubiquitylation in DDR. We hypothesized that the ubiquitylation of some of these proteins may induce their degradation and help to maintain cell viability upon MMS treatment. If such, overexpression of these proteins might increase cell sensitivity to MMS. To test this hypothesis, we transformed WT yeast cells with overexpression plasmids and assayed the sensitivity of transformed cells to MMS (Fig. 4). To this goal, we used the yeast ORF collection from Open Biosystems, a collection of 2-micron plasmid from which individual ORFs are expressed under the control of the galactose inducible *GAL1* promoter, which is repressed when glucose is the only source of carbon. As a control, we used overexpression of *HST3*, which is known to inhibit yeast growth in the presence of MMS. We compared the differences in MMS sensitivity of cells transformed with an empty vector (BG1805) *versus* cells bearing a plasmid with overexpressed genes (galactose medium). A glucose medium was used as a control of growth in the absence of gene overexpression. As expected, overexpression of *HST3* robustly inhibited the growth of MMS-treated cells (Fig. 4). Although overexpression of many of the potentially ubiquitylated proteins identified in our proteomic screen did not confer strong sensitivity to MMS, we identified several genes which had a clear effect. This was in particular the case for *POL30*, but also *SSL1*, *RFA2*, *RPC34*, and *PFS2*. Importantly, overexpression of several genes inhibited cell growth even in the absence of MMS, making it difficult to interpret the effect of MMS. This was for instance the case for the overexpression of *ASF1*, *UBX4*, *SIC1*, *ORC3*, *MRS6*, *MND2*, *FRK1*, *FUN30*, *PAB1*, and *SIP4*.

### Protein Abundance Analysis Upon MMS Treatment

**Fig. 5:**
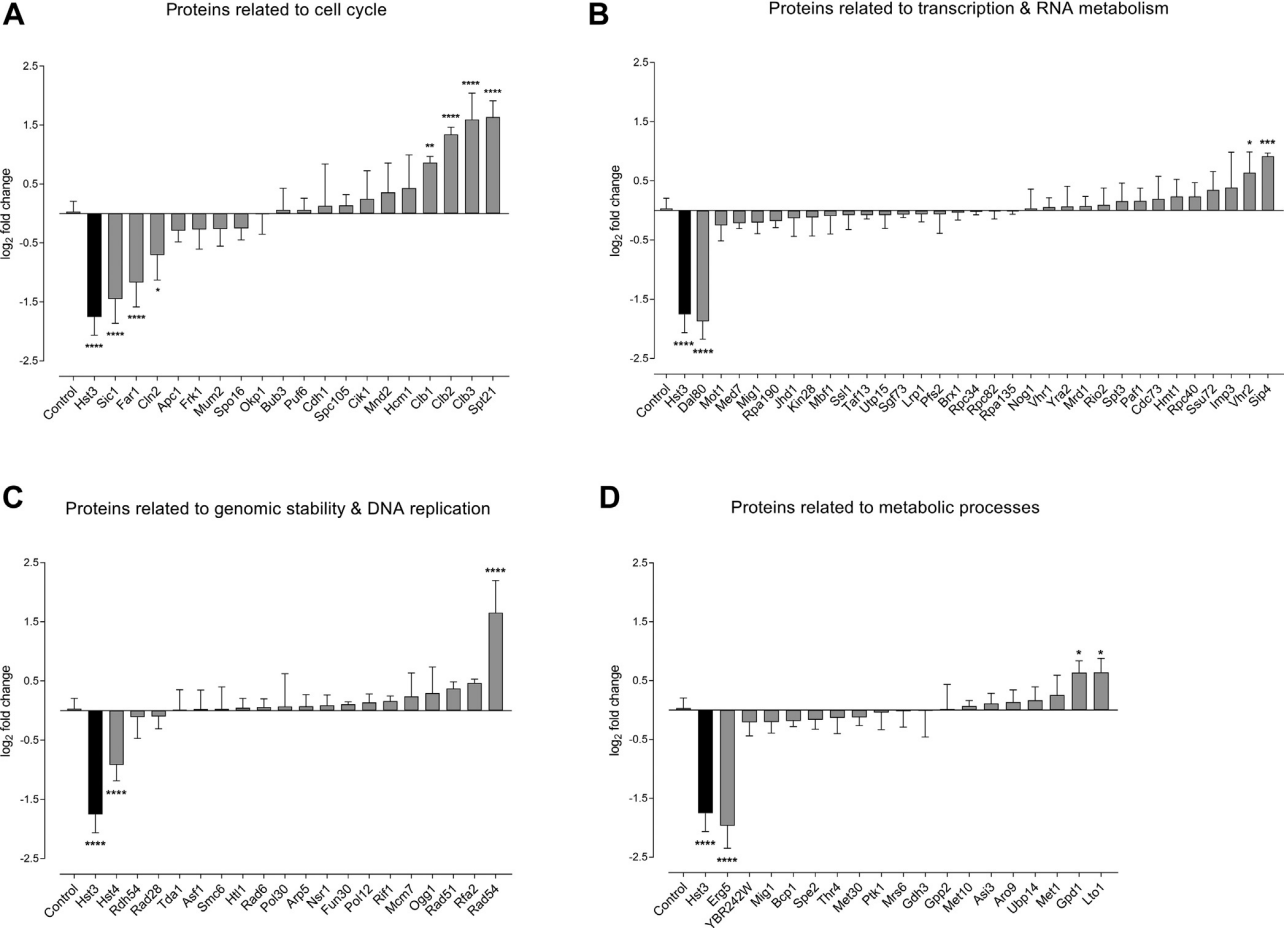
**Effect of MMS treatment onrelative protein abundancesmeasured using a NanoLuc reporter.***A*–*D*, the luminescence of yeast strains expressing the indicated proteins endogenously tagged with NanoLuc was measured 10 min and 2 h after treatment with 0.03% MMS. The histograms display the mean of log_2_ transformed luminescence intensity fold changes between both time points from at least three independent experiments. Error bars correspond to standard deviation. Hst3 (in *black*) was used as a control protein known to be degraded upon MMS treatment. Control (in *white; left side* close to the *y*-axis) indicates a group of proteins unexpected to be degraded upon MMS treatment (Rad5, Nup82, Nup85, Nup120, Nup133, Nup159, Nup188, Ubp11, Dyn2). The significance of the difference between each of the investigated proteins and the control group was determined by ordinary one-way ANOVA at the confidence level of 95% (∗∗∗∗ *p* < 0.0001, ∗∗∗ *p* < 0.002, ∗∗ *p* < 0.0021, and ∗ *p* < 0.0332) using the GraphPad Prism software. MMS, methyl methanesulfonate.

We next investigated the abundance of the proteins analyzed *via* gene overexpression as well as several other proteins identified or not in the proteomic screen (Fig. 5). We reasoned that the ubiquitylation of some of these proteins might induce their degradation. To probe their abundance, we took advantage of NanoLuc, a bright 19 kDa luciferase, with enhanced stability and low background activity that facilitates the monitoring of protein levels (66, 67). We constructed a collection of yeast strains expressing genes endogenously fused to the coding sequence of NanoLuc (unpublished). We selected from this collection 87 strains with mostly nuclear proteins. As controls, we used the Hst3-NanoLuc strain, since Hst3 is known to be degraded upon MMS treatment (26), and several NanoLuc-tagged proteins, which we expected to be stable upon MMS (mainly nucleoporins). NanoLuc activity was monitored 10 min and 2 h after MMS treatment (0.03%). Figure 5 shows the fold change (log_2_ scale) of the NanoLuc signal after MMS treatment for each strain relative to the mean of the negative control strains. Investigated proteins were grouped according to their function (cell cycle, transcription and RNA metabolism, genomic stability and DNA replication, and metabolic processes). As expected, we observed that MMS treatment induced a strong reduction of Hst3-NanoLuc levels. We also observed a statistically significant decrease in the abundance of the Hst3 paralog Hst4, the cell cycle regulators Sic1, Far1, and Cln2, as well as the transcription factor Dal80 and the ergosterol synthesis enzyme Erg5. Conversely, we observed some proteins, which displayed a significant increase in abundance following MMS treatment, such as the B-type cyclins (Clb1, Clb2, and Clb3), the cell cycle regulated protein Spt21, or the DNA repair protein Rad54. However, in most instances, the abundance of the investigated proteins was essentially unchanged. These results thus indicate that the majority of MMS-induced ubiquitylation events do not lead to a global proteolytic inactivation of the targeted proteins.

## Discussion

Ubiquitin plays a critical role in the DDR by regulating the localization, activity, and stability of DDR-associated proteins. The DDR is a vast signal-transduction network that responds to genomic lesions, promoting cell survival. To increase our understanding of the role of ubiquitylation in the DDR, we performed a proteomic screen to identify proteins that may be ubiquitylated upon MMS treatment in budding yeast. This screen was performed under conditions where the nuclear proteasome activity was largely diminished, thanks to the anchor-away system. We expected that this experimental strategy would facilitate the identification of nuclear proteins targeted for proteasomal degradation upon MMS treatment. Yet, we cannot exclude that some of the MMS-induced ubiquitylation targets that we identified are not directly involved in the DDR or may be subject to nonproteolytic ubiquitylation events. For instance, MMS treatment induces the accumulation of cells in S-phase (68), which may lead to changes in protein ubiquitylation profiles that are not directly related to the DDR. Indeed, we observed using the NanoLuc reporter that the abundance of several cell cycle–regulated proteins identified in our screen (Sic1, Far1, Cln2, Clb1-3, Spt21) varied upon MMS treatment (Fig. 5). Hence, changes in the ubiquitylation of these or other proteins whose activity or abundance is regulated during the cell cycle may be an indirect consequence of cell cycle perturbation. MMS is also well described to reprogram gene expression patterns (69, 70), which will also lead to alteration of protein abundances and ubiquitylation. For instance, MMS is known to induce the expression of the DNA repair protein Rad54 (71), a phenomenon which we also observed in our NanoLuc assay (Fig. 5). Thus, the possible increase in Rad54 ubiquitylation observed in MMS-treated cells (supplemental Table S3) may be an indirect effect of its higher expression level under those conditions. Last, it is possible that some proteins ubiquitylated upon MMS treatment are actually targeted by quality control mechanisms that eliminate damaged (methylated or oxidized) proteins.

Despite these considerations, our results corroborate and extend a large body of literature showing that ubiquitin plays a critical role in the orchestration of DDR pathways. For example, the yeast nuclear Pol30 protein (human PCNA), is well known to be ubiquitylated on various residues, depending on the nature of DNA lesions. Ubiquitylation on K164 leads to the recruitment of translesion polymerases that are able to replicate damaged DNA template (15, 16, 17). The identification of K210 as a new MMS-induced ubiquitylation site of Pol30 further documents the notion that DNA-damaging conditions can evoke multiple ubiquitylation events on different sites of yeast PCNA (35).

Our proteomic results enabled us to identify 435 proteins with 519 ubiquitylation sites potentially upregulated upon MMS treatment. An interesting candidate is replication factor A protein 2 (Rfa2). We found that Rfa2 is ubiquitylated on K199 in the MMS-treated condition only (supplemental Table S4) and that its overexpression increases MMS sensitivity (Fig. 4). Rfa2 is a subunit of the ssDNA-binding replication protein A (RPA) complex, which plays a fundamental role in DNA replication and repair (72, 73). RPA serves as a sensor of ssDNA regions, protects ssDNA from degradation, removes secondary structures, and acts as a platform to recruit DNA damage signaling and repair proteins to stalled replication forks or resected DNA ends (73, 74, 75). In human cells, RPA subunits are polyubiquitylated by the E3 RFWD3 in response to replication fork blocking agents, leading to the timely removal of RPA from DNA damage sites to promote replication fork repair by homologous recombination (76, 77, 78). In addition, RPA has also been reported to be polyubiquitylated by the U-box family ubiquitin ligase PRP19/PSO4, which is required for efficient recruitment of the DNA damage sensor kinase ATR to stalled replication fork and DNA damage signal transduction (79, 80). The yeast Rfa2 is known to be phosphorylated (81) and sumoylated upon MMS (82, 83). However, DNA damage-induced ubiquitylation of RPA and its role in replication stress response has yet to be reported in yeast cells. Interestingly, the yeast *PRP19/PSO4* gene, known primarily for its role in splicing of nuclear pre-mRNAs by the spliceosome (84), was originally isolated in a screen for mutations conferring radiosensitivity and has also been found to be required for resistance to 8-methoxypsoralen photoaddition, nitrogen mustard and UV radiation (85). DNA damage sensitivity of *pso4* mutants is generally attributed to the downregulation of intron-containing genes involved in DNA repair (86). It is therefore tempting to speculate that, as in mammals, the yeast Prp19/Pso4 might also regulate the DDR through RPA ubiquitylation.

Erg5 is another interesting candidate identified in our screen. We observed MMS-induced ubiquitylation of Erg5 at four lysine residues (K188, K424, K519, and K528) (supplemental Table S4). Furthermore, Erg5 abundance was strongly reduced in MMS-treated cells (Fig. 5). Erg5 is a cytochrome P450 desaturase involved in the late stages of ergosterol biosynthesis (87), which localizes at the endoplasmic reticulum and the nuclear periphery (88). Lipid metabolism at the inner nuclear membrane controls nuclear envelope functions and preserves nuclear envelope integrity (89). We observed that, in addition to Erg5, MMS induced the ubiquitylation of other enzymes of the ergosterol synthesis pathway, including Erg11, Erg27, Erg9, and Mvd1 (supplemental Table S4). Furthermore, previous studies described that ergosterol biosynthesis genes (including *ERG5*) are repressed in MMS-treated cells (69). This suggests that yeast cells have developed a coordinated response, which involves both transcriptional and proteolytic regulatory pathways, to control ergosterol biosynthesis in MMS-treated cells. Since MMS and other genotoxic agents induce lipid stress at the inner nuclear membrane in yeast and mammalian cells (90, 91), this response may serve to regulate lipid and nuclear homeostasis under certain genotoxic stress conditions.

Further studies will be needed to validate and then functionally investigate the MMS-induced ubiquitylation of Rfa2, Erg5 or other candidates identified in our screen. Importantly, our NanoLuc data indicate that in most instances ubiquitylation of these proteins may not lead to a global change in their turnover or abundance. Hence, ubiquitylation may either regulate their activity in a nonproteolytic manner or only affect a small fraction of their total cellular pool. Distinguishing between these hypotheses will require mechanistic studies to uncover the circumstances of their ubiquitylation and dissect the nature of their ubiquitin conjugates. In both cases, the datasets of ubiquitylated proteins and conjugation sites we have generated will be an instrumental resource for future research on both ubiquitylation and DDR pathways.

## Data Availability

The mass spectrometry proteomics data have been deposited to the ProteomeXchange Consortium *via* the PRIDE (92) partner repository with the dataset identifier PXD043291 and 10.6019/PXD043291.

## Supplemental data

This article contains supplemental data, including Supplementary Figures (31, 34, 40, 51, 61, 62, 63, 93, 94, 95, 96, 97, 98, 99, 100).

## Conflict of interest

The authors declare no competing interests.
