# Supplementary material for: Dissecting Ubiquitylation and DNA Damage Response Pathways in the Yeast Saccharomyces cerevisiae Using a Proteome-Wide Approach
Source: Mol Cell Proteomics. 2023 Dec 14;23(1):100695. doi: 10.1016/j.mcpro.2023.100695 (PMC10803944; doi:10.1016/j.mcpro.2023.100695)
Supplement: Table S5 [file mmc5.pdf]

Table S5. Selection of terms used for the gene ontology analysis.

This selection was based on list of 164 predefined GO slims restricted to the yeast taxonomy (https://www.ebi.ac.uk/QuickGO/slimming; accessed: May 2023, revised: November 2023)

Domain	Gene Ontology term
Cellular Component (CC)	GO:0005634 – nucleus<br>GO:0005694 – chromosome<br>GO:0005730 – nucleolus<br>GO:0005737 – cytoplasm<br>GO:0005739 – mitochondrion<br>GO:0005773 – vacuole<br>GO:0005783 – endoplasmic reticulum<br>GO:0005794 – Golgi apparatus<br>GO:0005840 – ribosome<br>GO:0005886 – plasma membrane
Biological Process (BP)	GO:0000278 – mitotic cell cycle<br>GO:0006281 – DNA repair<br>GO:0006325 – chromatin organization<br>GO:0006457 – protein folding<br>GO:0006260 – DNA replication<br>GO:0006310 – DNA recombination<br>GO:0006352 – DNA-templated transcription initiation<br>GO:0006353 – DNA-templated transcription termination<br>GO:0006354 – DNA-templated transcription elongation<br>GO:0006360 – transcription by RNA polymerase I<br>GO:0006366 – transcription by RNA polymerase II<br>GO:0006383 – transcription by RNA polymerase III<br>GO:0006417 – regulation of translation<br>GO:0006974 – DNA damage response<br>GO:0006997 – nucleus organization<br>GO:0007059 – chromosome segregation<br>GO:0016570 – histone modification<br>GO:0023052 – signaling<br>GO:0031399 – regulation of protein modification process<br>GO:0042221 – response to chemical<br>GO:0051052 – regulation of DNA metabolic process<br>GO:0051169 – nuclear transport<br>GO:0051726 – regulation of cell cycle<br>GO:0070647 – protein modification by small protein conjugation or removal
Molecular Function (MF)	GO:0003677 – DNA binding<br>GO:0003682 – chromatin binding<br>GO:0003700 – DNA-binding transcription factor activity<br>GO:0003723 – RNA binding<br>GO:0003729 – mRNA binding<br>GO:0004386 – helicase activity<br>GO:0004518 – nuclease activity

	GO:0008134 – transcription factor binding<br>GO:0016874 – ligase activity<br>GO:0019899 – enzyme binding<br>GO:0030234 – enzyme regulator activity<br>GO:0032182 – ubiquitin-like protein binding<br>GO:0042393 – histone binding<br>GO:0140657 – ATP-dependent activity
