# Supplementary material for: Dissecting Ubiquitylation and DNA Damage Response Pathways in the Yeast Saccharomyces cerevisiae Using a Proteome-Wide Approach
Source: Mol Cell Proteomics. 2023 Dec 14;23(1):100695. doi: 10.1016/j.mcpro.2023.100695 (PMC10803944; doi:10.1016/j.mcpro.2023.100695)

Figure S1. Subcellular localization of K48-linked ubiquitin chains in Pre8 and Sts1 anchor-away strains.

Control (HHY168), Sts1-FRB-GFP (scGR1123) and Pre8-FRB-GFP (scGR1115) anchor-away strains were treated or not with rapamycin for 16 h and then processed for immunofluorescence staining with an anti-K48-linked ubiquitin antibody and DAPI (to visualize the nucleus). In the merged images, the anti-K48 staining is shown in red and the DAPI staining in blue.

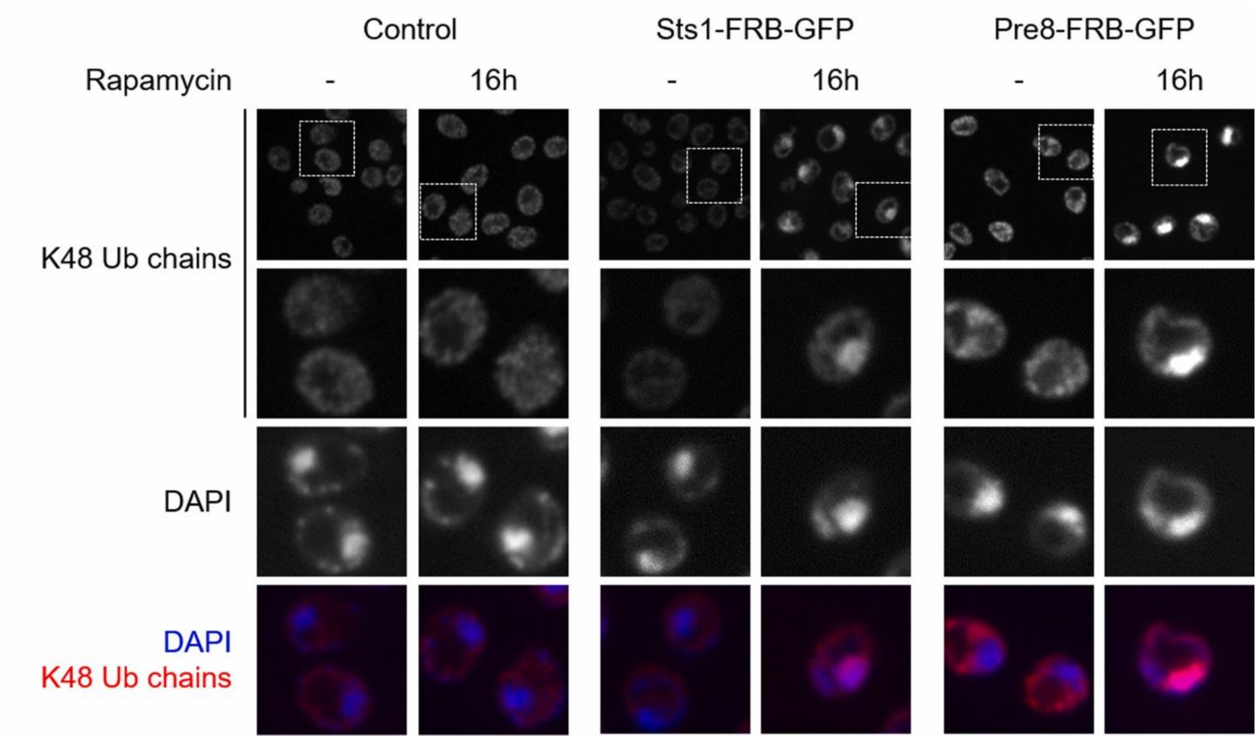

Supplement: Figure S1 [file mmc7.pdf]
