# Supplementary material for: Dissecting Ubiquitylation and DNA Damage Response Pathways in the Yeast Saccharomyces cerevisiae Using a Proteome-Wide Approach
Source: Mol Cell Proteomics. 2023 Dec 14;23(1):100695. doi: 10.1016/j.mcpro.2023.100695 (PMC10803944; doi:10.1016/j.mcpro.2023.100695)

Figure S2. Effect of the expression of exogenous His-ubiquitin in the Pre8 anchor-away strain.

A-B. Immunoblots of total protein extracts prepared from the Pre8-FRB-GFP anchor-away strain (scGR1115) transformed with an empty vector (-) (pGR135) or a His-ubiquitin expressing plasmid (+) (pGR295). The membranes were probed with anti-total ubiquitin (P4D1) (A) or K48-linked ubiquitin (Apu2) (B) antibodies. Ponceau staining was used to control equal protein loading.

C. MMS sensitivity of wild type (WT) (scGR1115) and *UBC13* deleted (*ubc13Δ*) (scGR1306) Pre8-FRB-GFP strains (0.012%) transformed with an empty vector (-) (pGR135) or a His-ubiquitin expressing plasmid (+) (pGR295). The transformants were serially diluted and spotted on SC medium plates (without leucine) supplemented with and without MMS. The *ubc13Δ* mutant strain was used as a control showing increased sensitivity to MMS.

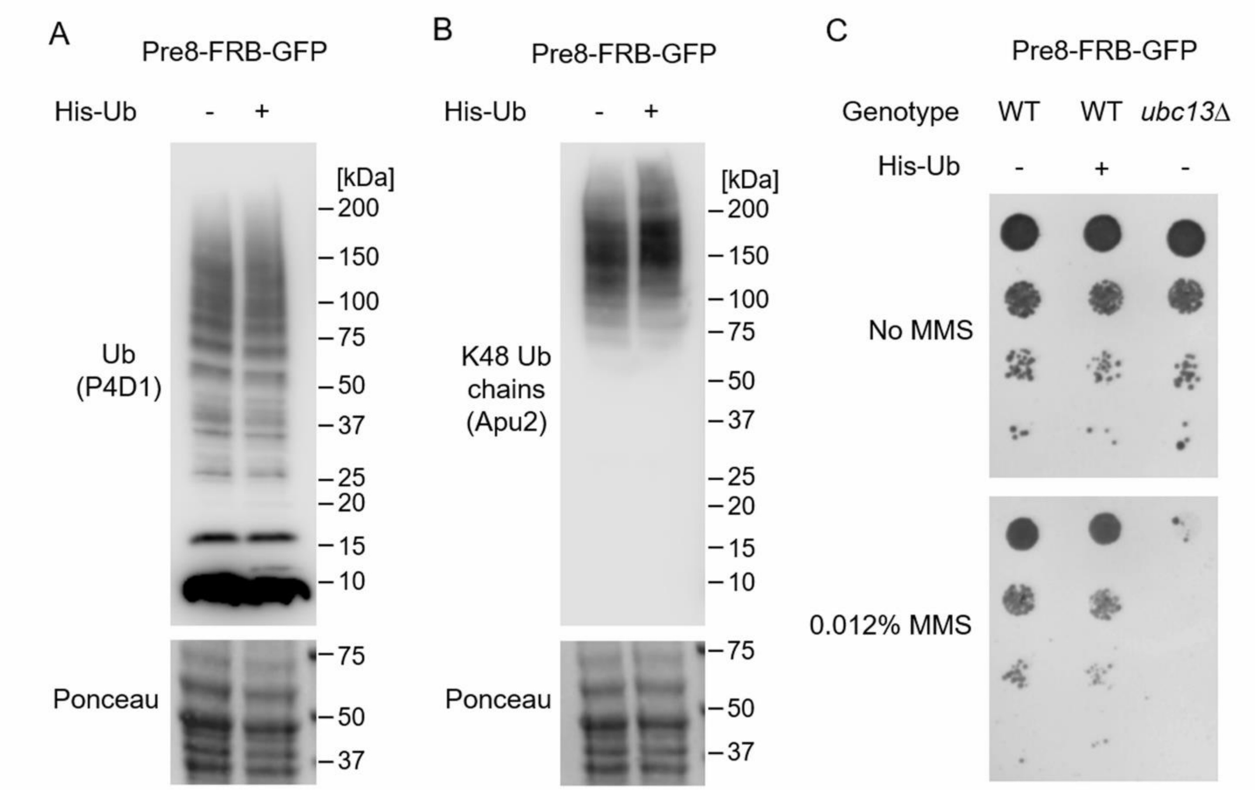

Supplement: Figure S2 [file mmc8.pdf]
